# Supplementary material for: Objects guide human gaze behavior in dynamic real-world scenes
Source: PLoS Comput Biol. 2023 Oct 26;19(10):e1011512. doi: 10.1371/journal.pcbi.1011512 (PMC10602265; doi:10.1371/journal.pcbi.1011512)
Supplement: S1 Table — The column “Subjects” shows the number of human observers, each contributing to ground truth scanpaths in the human data, and “Split” indicates if the video was used in the training or test set. (PDF) [file pcbi.1011512.s001.pdf]

Table S1. List of the 23 videos of the VidCom dataset used in this study. The column "*Subjects*" shows the number of human observers, each contributing to ground truth scanpaths in the human data, and "*Split*" indicates if the video was used in the training or test set.

Name	Subjects	Split
dance01	12	train
dance02	14	train
field03	12	test
foutain02	14	test
garden04	12	test
garden06	14	train
garden07	13	train
garden09	12	test
park01	12	train
park06	12	train
park09	11	test
road02	11	train
road04	11	test
road05	11	train
robarm01	12	test
room01	10	train
room02	12	test
room03	10	test
tommy02	13	test
uscdog01	12	test
walkway01	13	test
walkway02	13	test
walkway03	13	train
