# Supplementary figures and images for: Objects guide human gaze behavior in dynamic real-world scenes
Source: PLoS Comput Biol. 2023 Oct 26;19(10):e1011512. doi: 10.1371/journal.pcbi.1011512 (PMC10602265; doi:10.1371/journal.pcbi.1011512)

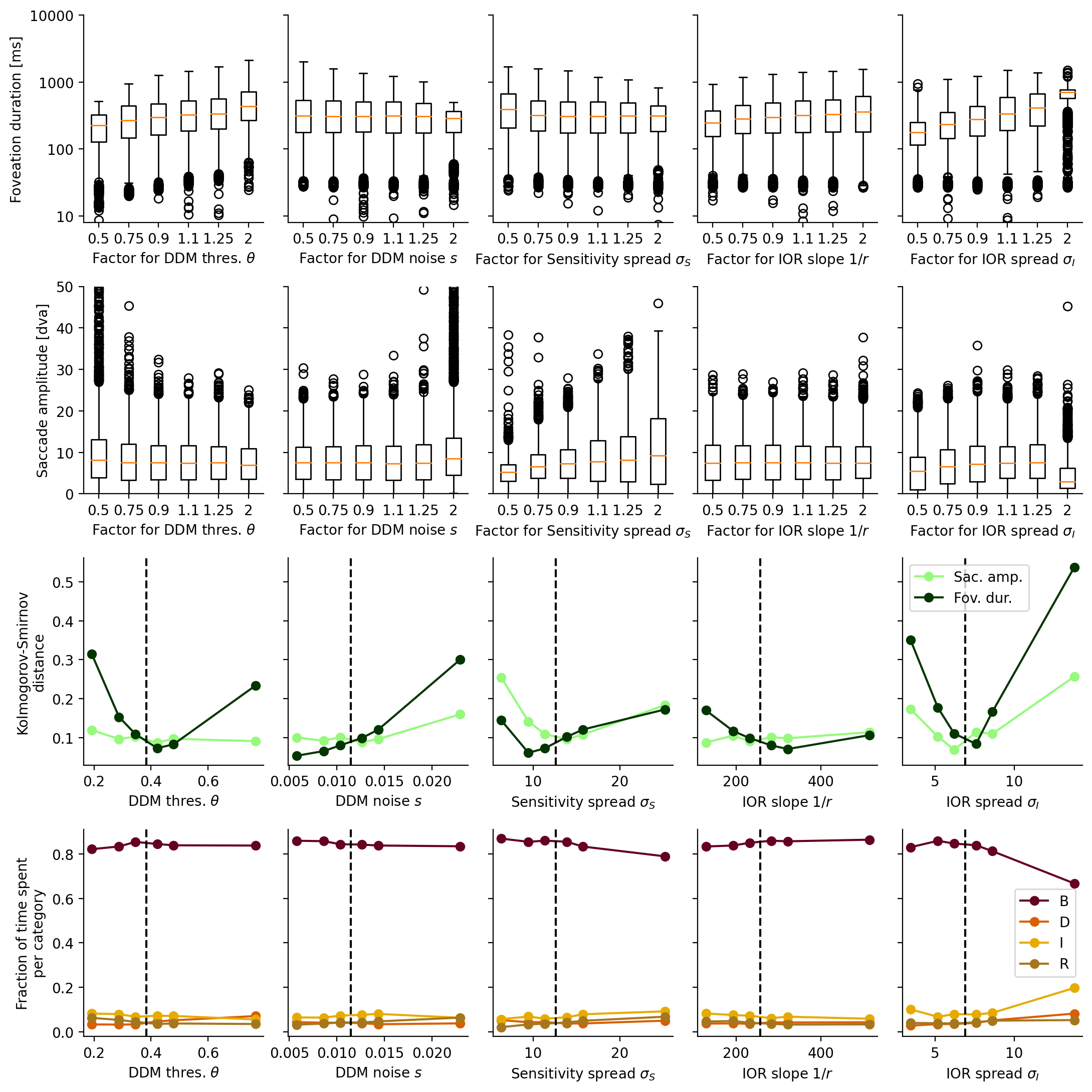

Supplement: S1 Fig — We use the mean parameter values from the last generation of the evolutionary algorithm, as reported in Table 1, as default parameters (indicated by the dashed line in the third and fourth rows). We then vary each parameter individually by multiplying with the factor 0.5, 0.75, 0.9, 1.1, 1.25, or 2. With all other parameters set to the default value, we simulate twelve scanpaths for each video in the VidCom dataset for each factor. From the resulting scanpaths, we plot the foveation duration (first row) and the saccade amplitude (second row) summary statistic as box plots, the resulting fitness measured by the respective KS-statistic (third row), and the fraction of stimulus time spent in each of the four foveation categories (fourth row). (TIF) [file pcbi.1011512.s002.tif]

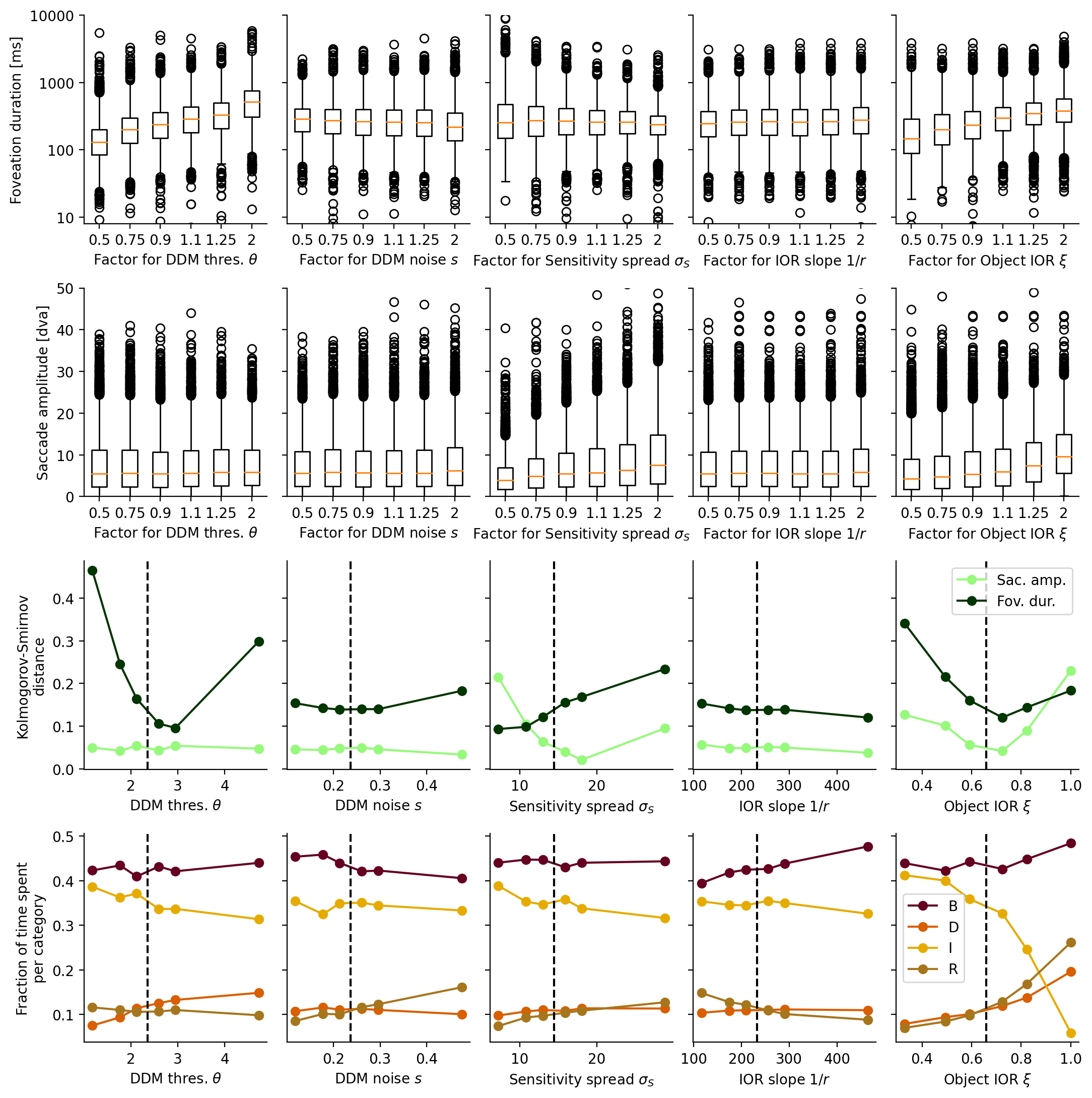

Supplement: S2 Fig — We use the mean parameter values from the last generation of the evolutionary algorithm, as reported in Table 2, as default parameters (indicated by the dashed line in the third and fourth rows). We then vary each parameter individually by multiplying with the factor 0.5, 0.75, 0.9, 1.1, 1.25, or 2 (the object-based inhibition parameter ξ ∈ [0, 1] is set to ξ = 1 for factor 2). With all other parameters set to the default value, we simulate twelve scanpaths for each video in the VidCom dataset for each factor. From the resulting scanpaths, we plot the foveation duration (first row) and the saccade amplitude (second row) summary statistic as box plots, the resulting fitness measured by the respective KS-statistic (third row), and the fraction of stimulus time spent in each of the four foveation categories (fourth row). (TIF) [file pcbi.1011512.s003.tif]

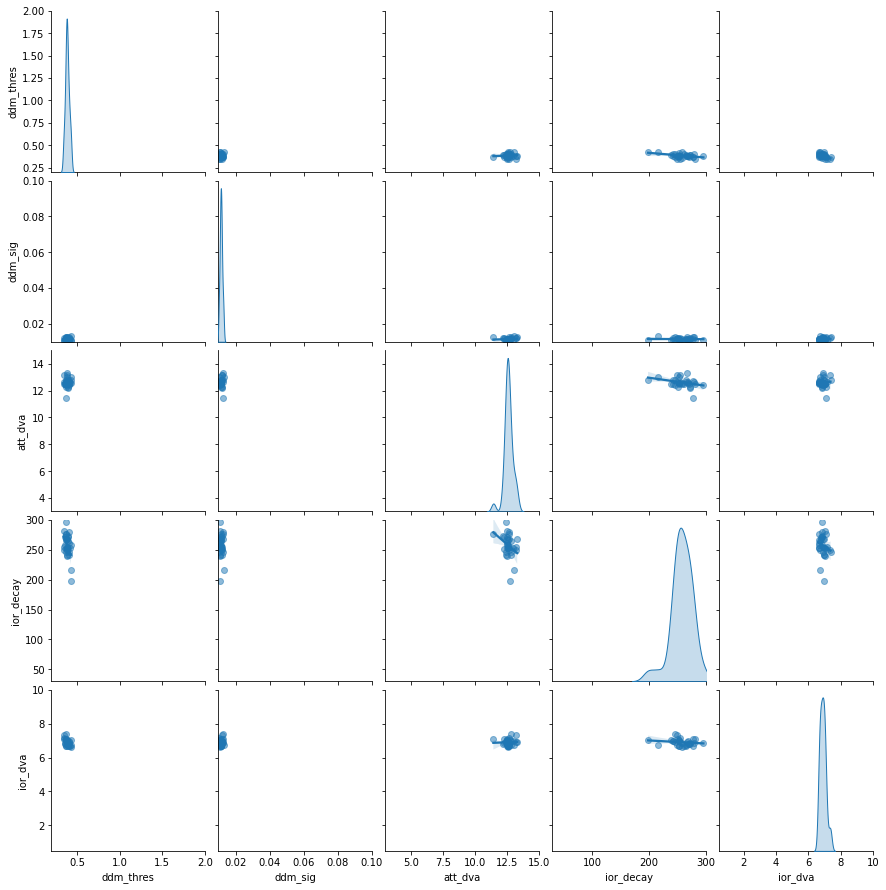

Supplement: S3 Fig — The diagonal shows for each parameter the distribution across the last generation of the evolutionary optimization process. The last generation contains the 32 model parameter configurations with the highest fitness (as defined in Eq (8)). Other panels show how pairs of parameters relate to each other. (TIF) [file pcbi.1011512.s004.tif]

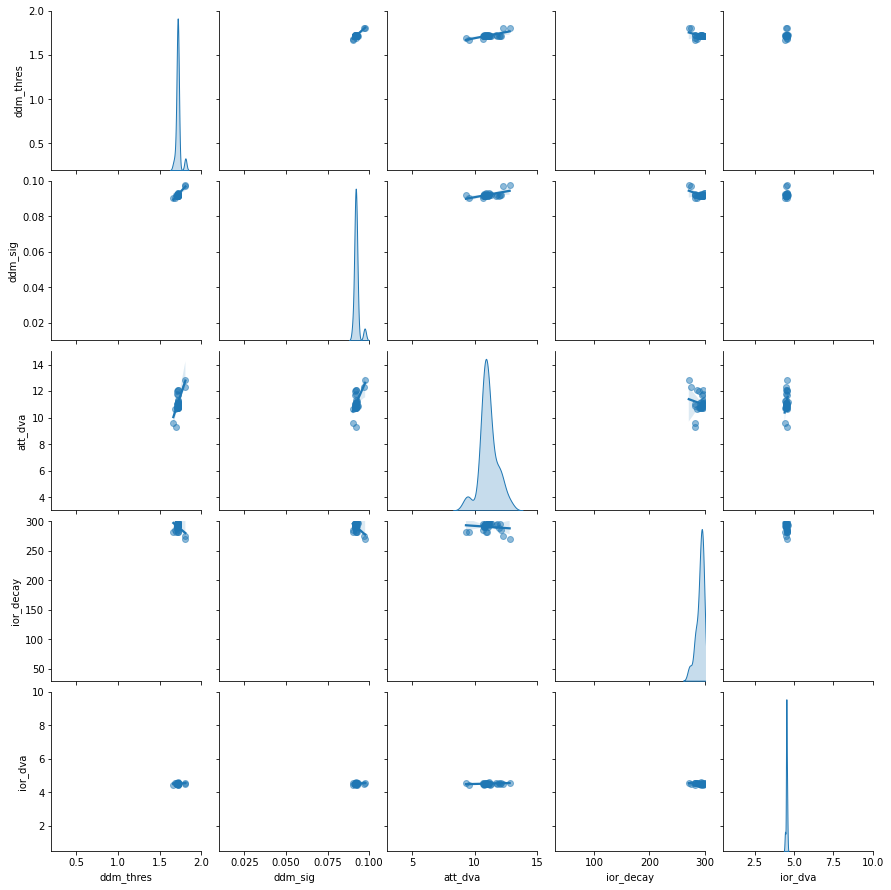

Supplement: S4 Fig — (TIF) [file pcbi.1011512.s005.tif]

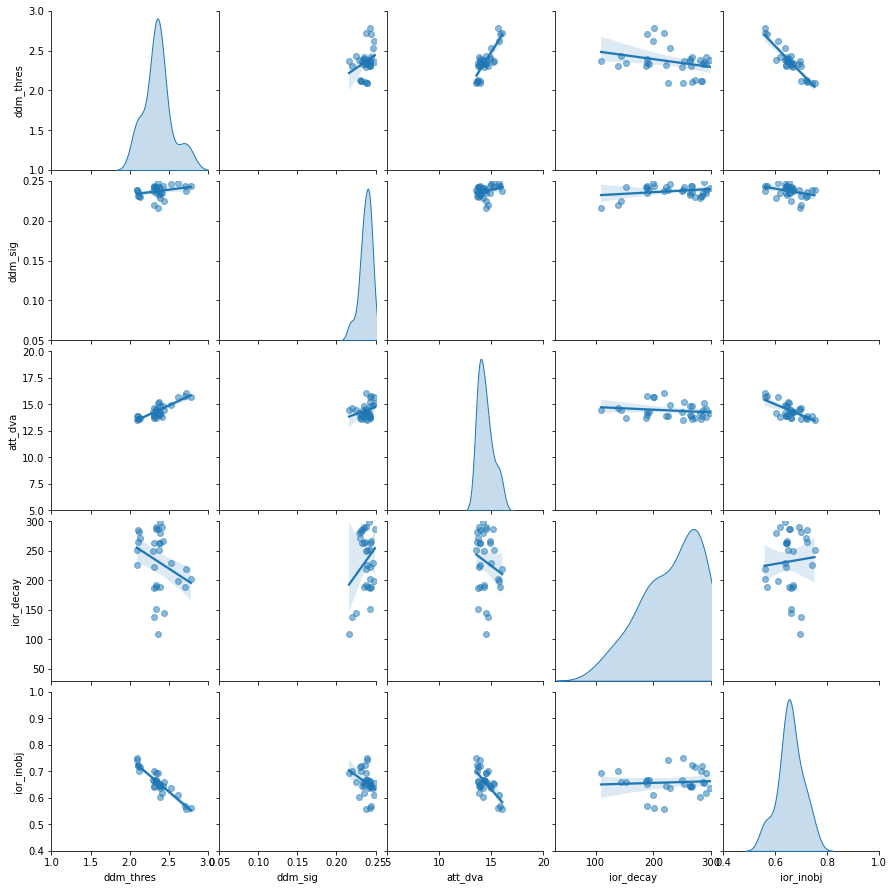

Supplement: S5 Fig — (TIF) [file pcbi.1011512.s006.tif]

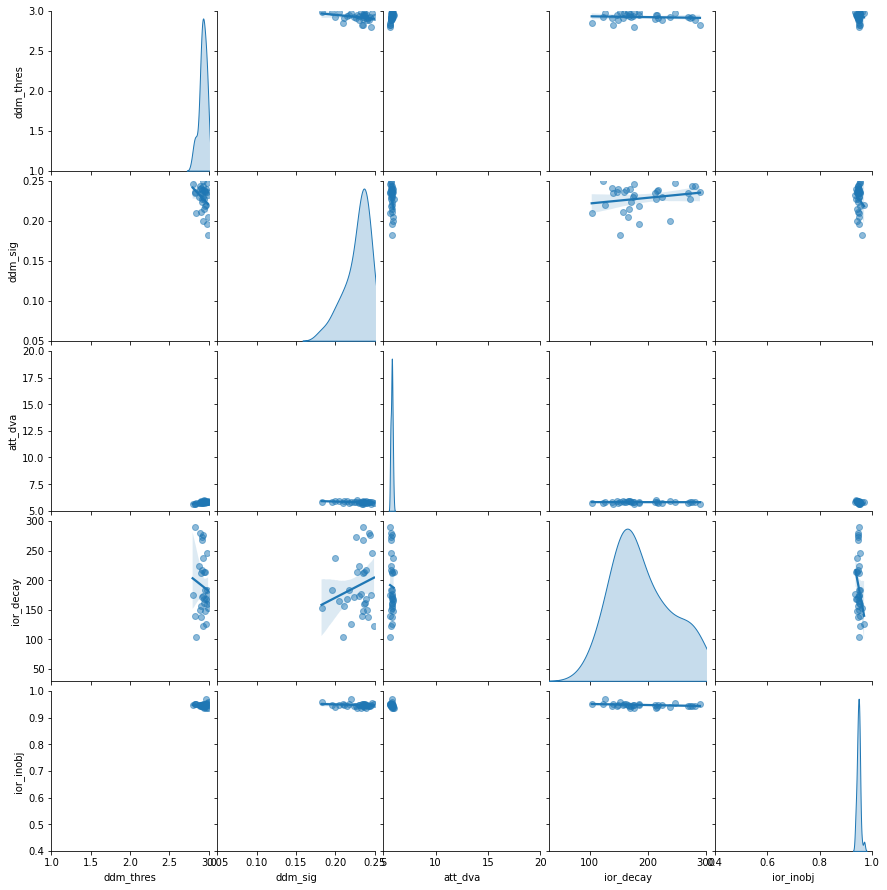

Supplement: S6 Fig — (TIF) [file pcbi.1011512.s007.tif]

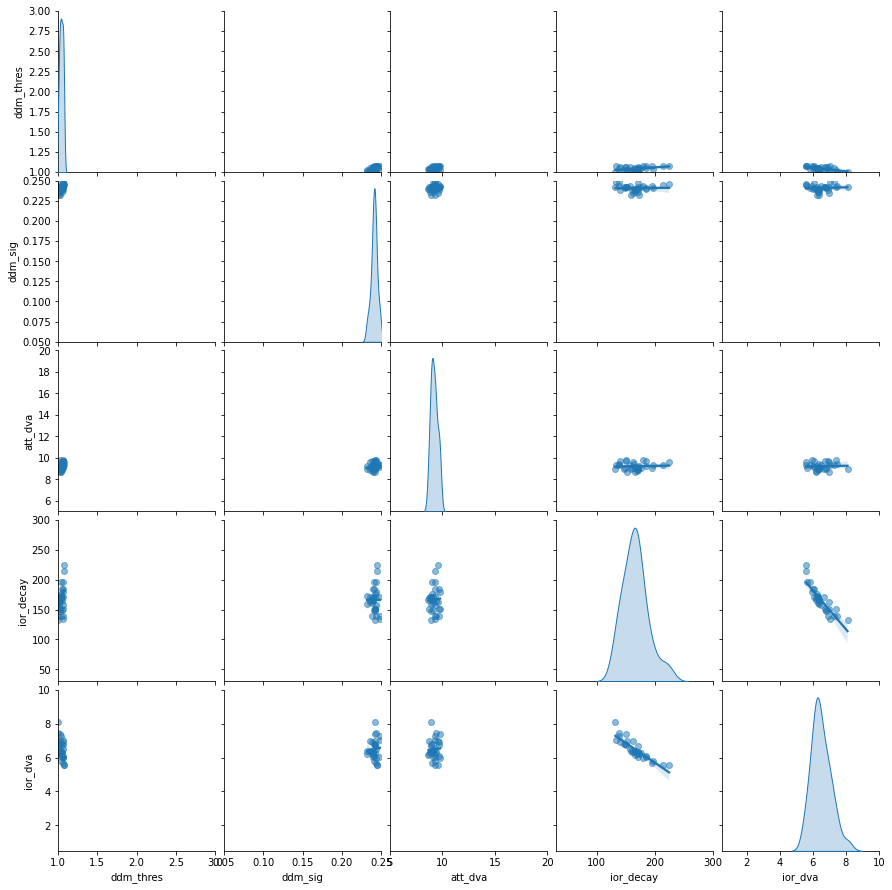

Supplement: S7 Fig — (TIF) [file pcbi.1011512.s008.tif]

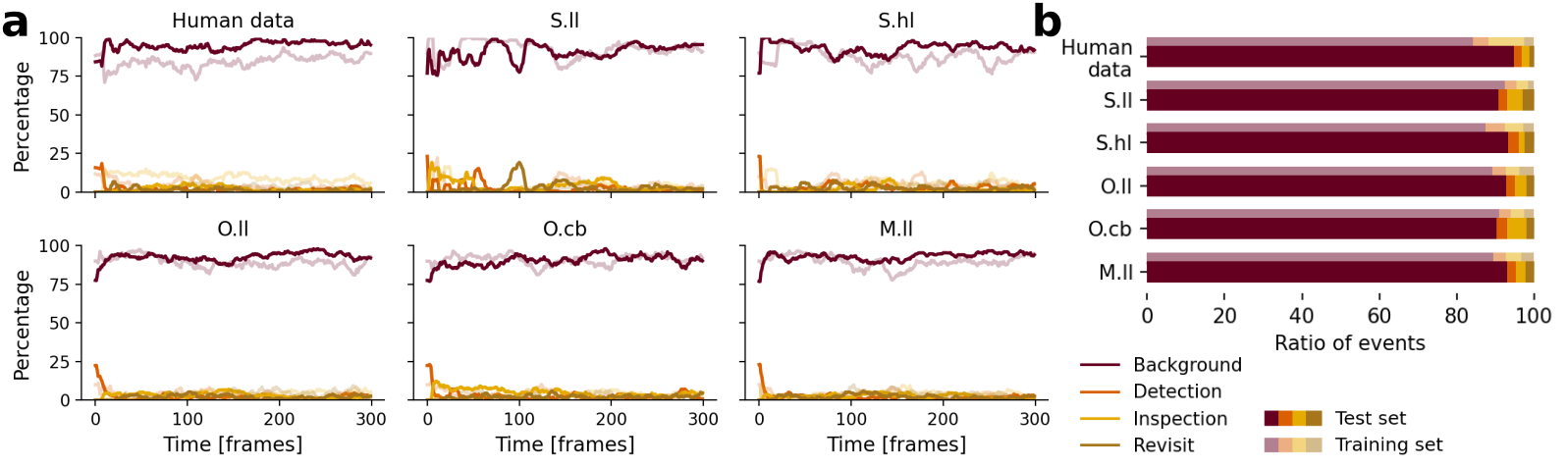

Supplement: S8 Fig — While the scanpaths from both the models and human observers remain unchanged, the corresponding video was reversed in time and mirrored on the x-axis and the y-axis. This manipulation significantly disrupts the correlation between scanpaths and objects in the scenes. Given that the majority of scene areas correspond to the general background category, we expected an extensive “exploration” of the background. (The relatively reduced background exploration time observed in human training data can be attributed to two videos featuring an object centrally positioned within the scene.). (TIF) [file pcbi.1011512.s009.tif]

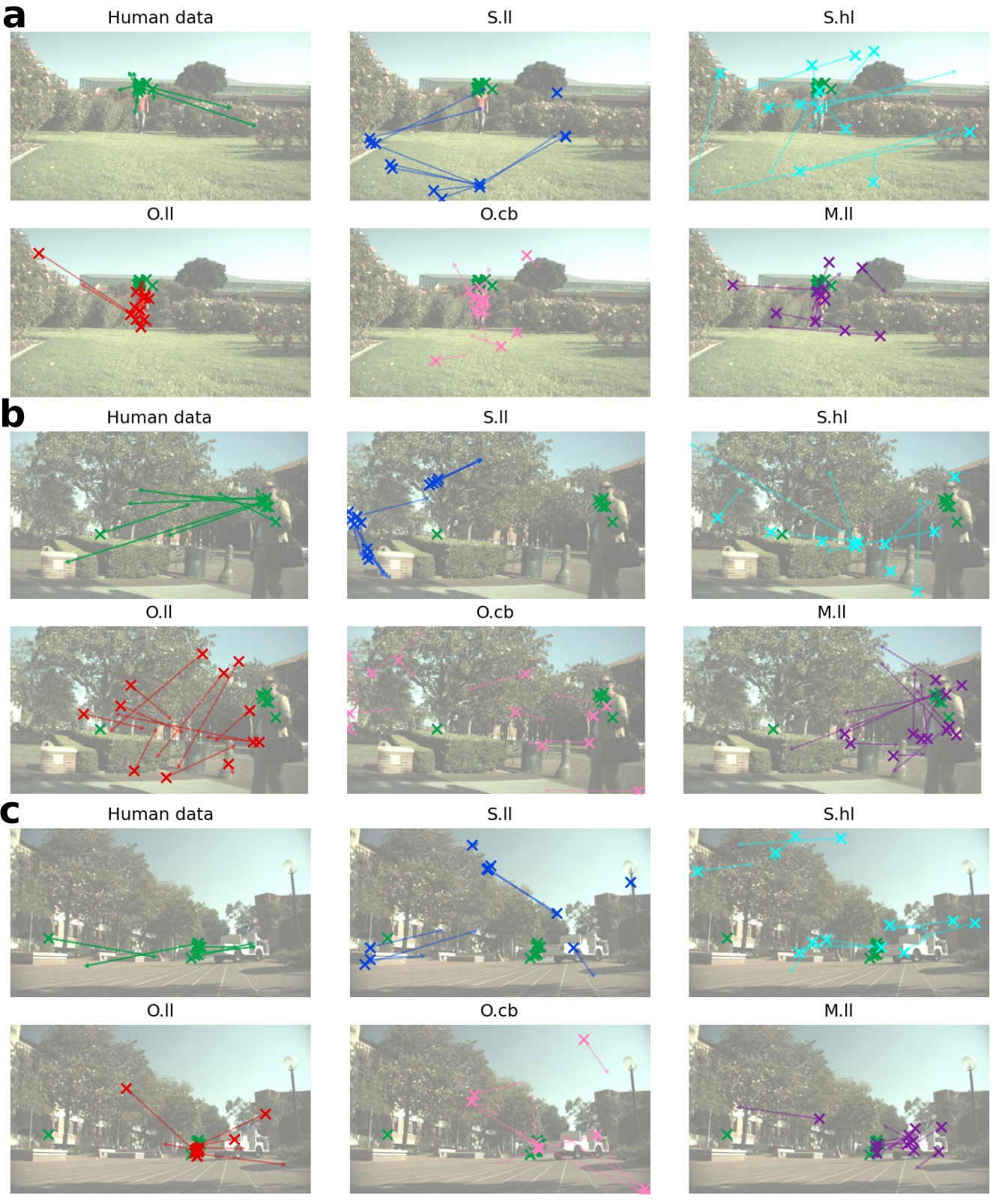

Supplement: S9 Fig — We identify prominent peaks in inter-observer consistency (measured by Normalized Scanpath Saliency (NSS) score as in [26]) and select three representative examples from the test set. We plot the current gaze position for the given frame for all observers and model runs and an arrow to the next saccade target. (Note that this might not reflect the actual trajectory due to smooth pursuit before the saccade.) The current gaze data of humans (in green) is plotted in all panels for comparison. (a) Video park09, frame 60. This example shows how object-based models tend to select the correct object but do not replicate the strong tendency of looking at faces observed in humans. (b) Video garden04, frame 177. This is a failure case of the O.ll model, where not yet enough evidence is accumulated to select the object which newly entered the scene. (c) Video walkway01, frame 262. (TIF) [file pcbi.1011512.s010.tif]

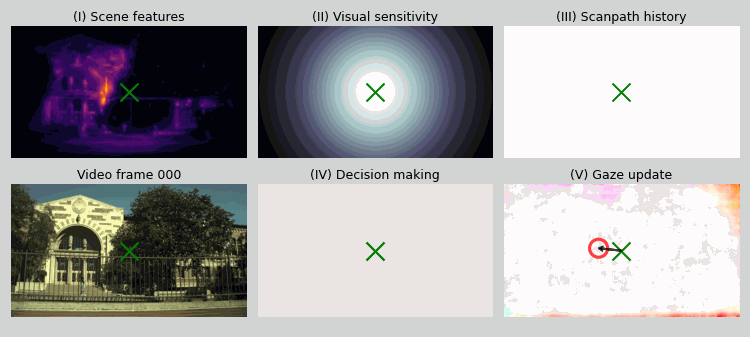

Supplement: S1 File — Animation of the simulated gaze position (green cross) on top of visualizations of the different modules of the space-based model with low-level features (S.ll). The bottom left panel shows the original field03 video. (I) Precomputed low-level saliency map with anisotropic center bias. Low values are shown in dark, high values in bright colors. (II) Gaze dependent Gaussian visual sensitivity map. Black means not sensitive (0), white means fully sensitive (1). (III) Inhibition of return map (value calculated for every pixel). White means no inhibition (0), black means fully inhibited (1). (IV) Visualization of the decision variable of each pixel-location. The saturation of a pixel represents the amount of accumulated evidence (white corresponds to 0, dark red to the decision threshold θ). (V) The red circle indicates the next gaze position. The pixel values indicate the optical flow. (GIF) [file pcbi.1011512.s011.gif]

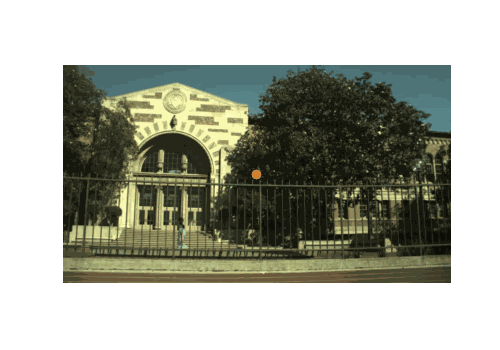

Supplement: S2 File — Animation of twelve simulated scanpaths of the space-based model with low-level features (S.ll) on the field03 video. Colors correspond to different random seeds when running the model with the parameter configuration with the highest fitness (see Table 1) and dotted lines indicate saccades. (GIF) [file pcbi.1011512.s012.gif]

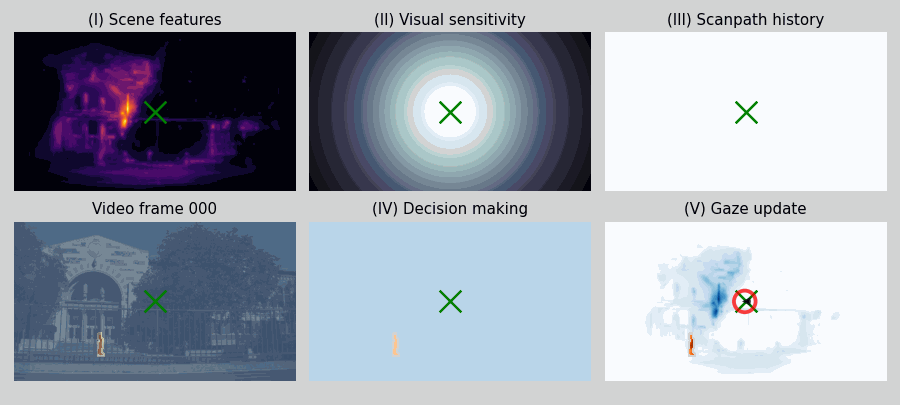

Supplement: S3 File — Animation of the simulated gaze position (green cross) on top of visualizations of the different modules of the object-based model with low-level features (O.ll). The bottom left panel shows the original field03 video. (I) Precomputed low-level saliency map with anisotropic center bias. Low values are shown in dark, high values in bright colors. (II) Gaze dependent visual sensitivity map, Gaussian with a uniform spread across currently foveated objects. Black means not sensitive (0), white means fully sensitive (1). (III) Visualization of the inhibition of return value of each object (attribute of the ObjectFile instance). White means no inhibition (0), black means fully inhibited (1). (IV) Visualization of the decision variable of each object (attribute of the ObjectFile instance). The saturation of the object mask represents the amount of accumulated evidence (white corresponds to 0, dark blue/red/green to the decision threshold θ). (V) The red circle indicates the next gaze position. The pixel values indicate for each object how likely each position within each object is as a saccade target (calculated from the features (I) and sensitivity (II)). (GIF) [file pcbi.1011512.s013.gif]

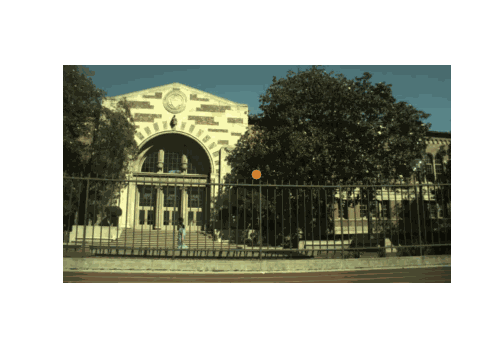

Supplement: S4 File — Animation of twelve simulated scanpaths of the object-based model with low-level features (O.ll) on the field03 video. Colors correspond to different random seeds when running the model with the parameter configuration with the highest fitness (see Table 2) and dotted lines indicate saccades. (GIF) [file pcbi.1011512.s014.gif]
