# Supplementary material for: Objects guide human gaze behavior in dynamic real-world scenes
Source: PLoS Comput Biol. 2023 Oct 26;19(10):e1011512. doi: 10.1371/journal.pcbi.1011512 (PMC10602265; doi:10.1371/journal.pcbi.1011512)
Supplement: Attachment — Submitted filename: Letter PCOMPBIOL-D-23-00435.pdf [file pcbi.1011512.s015.pdf]

Manuscript: PCOMPBIOL-D-23-00435

Responses to reviewers' comments

We sincerely thank the reviewers for their detailed feedback and valuable suggestions to our manuscript "Objects guide human gaze behavior in dynamic real-world scenes". We have significantly revised the manuscript accordingly. Below, we summarize the major revisions:

- We updated the phrasing throughout the manuscript to reflect precisely our goal of investigating the effects of object-based attentional mechanisms on human gaze behavior through mechanistic scanpath simulation in dynamic real-world scenes. This includes renaming the location-based models into *space*-based models to highlight their underlying assumption of space-based allocation of attention (in contrast to the object-based attention assumed in the object-based models).
- We incorporated saccade durations in our modeling framework. This led to small changes in the fitted parameters and numeric values in the results but did not change their interpretation.
- We provided a more detailed motivation for the choice of models compared within this framework at the end of the introduction and extended the explanation of the exact difference between model implementations at the beginning of the result section.
- We added a fifth "mixed model" which combines object-based attention and selection with space-based inhibition of return (IOR) and allows for a mechanistic investigation of IOR-related phenomena.
- We extended our results with an additional section called "*Sequential scanpath evaluation*", where we investigate the prioritization between objects, the number of return saccades, distributions of saccade turning angles and their effect on foveation time, and object-based return saccades.
- We complemented our functional scanpath evaluation with a comparison of the total dwell time on individual objects in the human data with the simulated scanpaths.
- We significantly expanded the Supplemental Material by adding a qualitative analysis of epochs with high inter-observer consistency and a control condition where we evaluate the scanpaths based on altered scene content.
- We extended the discussion to incorporate the results from the newly introduced sequential scanpath analysis and the mixed model, where we analyzed IOR-related behavior in detail. Additionally, we expanded the discussion on top-down guidance and connected our results more closely with previous findings of attention acting on objects with prioritization through salience in response to reviewers' comments.

In the following, we provide detailed responses (in black) along with the original comments (in blue and italic font). The line numbers are referring to the updated version of the manuscript, with changes in the "Revised Article with Changes Highlighted" in dark red.

Reviewer #1: This manuscript has many interesting bits and pieces but it does not have a coherent story.

Reading the abstract I stumbled over "Historically, eye movements were believed to be driven primarily by bottom-up saliency, but increasing evidence suggests that objects also play a significant role in guiding attention. " The view of an object is also bottom-up, stimulus driven. I view objects just as super features and not qualitatively different from other features. In the whole manuscript there is nothing about task / top-down guidance of eye movements (as is

admitted later in the discussion). It is not just a isolated glitch, the topic reoccurs in lines 116 following and lines 174 following. This confuses with respect to the topic of the study. Precise phrasing throughout would be super helpful.

The contrast we want to emphasize here is not bottom-up versus top-down guidance. Instead, the subject of this study is to use a new computational framework to investigate whether human gaze behavior is primarily guided through locations with salient features (space-based attention) or through objects (object-based attention). Importantly, objects are here not seen in terms of the features they might contribute to a saliency map but as units of perceptual organization on which attention operates. This is directly reflected in the computational architecture of the object-based models, where the object information does not enter the scene features (module I) but influences the attentional mechanisms in all other modules (more details in response to the next comment). We now also stress this in the first paragraph of the *Introduction,* where we added in line 37:

> *"We use this framework to quantitatively investigate the influence of object-based attention, which has been extensively investigated experimentally (e.g., Duncan 1984; Egly, Driver, and Rafal 1994; O'Craven, Downing, and Kanwisher 1999; for reviews see Scholl 2001; Cavanagh et al. 2022), in contrast to space-based attention on human gaze behavior in dynamic real-world scenes."*

We carefully revised the manuscript to avoid confusion about what we mean by objects guiding attention. This includes renaming the location-based models to space-based models. We changed the wording in the abstract accordingly to:

> *"Historically, eye movements were believed to be driven primarily by space-based attention towards locations with salient features. Increasing evidence suggests, however, that visual attention does not select locations with high saliency but operates on attentional units given by the objects in the scene."*

Other rephrasings include changing the name of the location-based models to *space*-based to better reflect the comparison between space-based and object-based attention in the literature and changing the title of the subsection in line 190 (formally line 174) from "Objects" to "Objects *as perceptual units*", where we now specify:

> *"The space-based models are only influenced by objects in the scene as far as the saliency map captures object-related features. In the object-based models, on the other hand, objects are the fundamental building blocks for the implementation of attention mechanisms and modulate the Visual sensitivity (II), Inhibition of return (III), Decision making (IV), and Gaze update (V) processes. [...]"*

An important aspect of the study is the mechanistic model of guiding eye movements. That's interesting. It suffers somewhat from the suggested contrast of location based and object based models. As far as I understand it, it is all location based. Only two models get an extra object recognition neural network and segmentation algorithm. Well, a pure DNN can recognise objects as well and does a good job at predicting fixations on objects (e.g. DeepGaze 3). So, what exactly are the important differences between L.hl and O.hl? Is it that the network has to provide precise segmentation boundaries to guide fixations precisely on the objects, in contrast to the center of gravity of objects? Is O.hl just much better at detecting objects than L.hl?

The distinction between the location and object-based models is crucial to the storyline of this manuscript, and we regret that the differences between the two model families did not become

clear in the original submission. We now describe the difference between the models in more detail at the beginning of the result section starting in line 462:

> *"Our first model S.ll is space-based and uses low-level feature information (Molin, Etienne-Cummings, and Niebur, 2015) quantifying concepts from saliency and the feature integration theory (Treisman and Gelade, 1980) of visual attention (see Fig. 1 with a, I, left). We primarily compare S.ll with the object-based model O.ll, which uses the same low-level saliency as scene features in module (I), but differs in all other modules (II-V) by the influence of objects on the attentional mechanisms (see Fig. 2 with a, I, left). While the visual sensitivity (module II) is Gaussian in S.ll, it additionally spreads evenly across the foveated object in O.ll, accounting for the spread of object-based attention (Egly, Driver, and Rafal, 1994; Malcolm and Shomstein, 2015; Scholl, 2001). The inhibition of previous saccade targets (module III) moves with the objects in O.ll (Tipper, Driver, and Weaver, 1991; Reppa and Leek, 2003) compared to the Gaussian inhibition of previous target locations in allocentric coordinates in S.ll. The decision-making process (module IV) uses object masks as potential targets for saccadic selection (Nuthmann and Henderson, 2010; Pajak and Nuthmann, 2013) and tracks the objects when updating the gaze position (module V) in O.ll, while S.ll selects individual pixel locations as saccade targets and updates the gaze with the optical flow to enable smooth pursuit behavior.*
>
> *To account for the possibility that visual attention and target selection are, in fact, space-based but that the low-level features used in the first model do not allow for a sufficiently good saliency prediction, we introduce a second space-based model S.hl where the model architecture is identical to S.ll but that uses high-level saliency as predicted by TASED-Net (Min and Corso, 2019) see Fig. 1 with a, I, right). [...]"*

Although we utilize DNNs to compute the high-level saliency maps, the optic flow for the gaze update in the space-based models, and to compute object segmentations and support tracking in the object-based models, these are all pre-computed and no DNN was trained for this study. Our models are fully mechanistic, which makes it possible to contrast a model where object information is only contained in the scene features (in the case of *L/S.hl*) with a model where objects are incorporated as perceptual units into attentional mechanisms (as in *O.ll*). (As there is no *O.hl* model in the manuscript, we assume that the reviewer refers to model *O.ll*.) We now refer to this comparison explicitly in the Discussion, starting in line 875:

> *"[...] The O.ll model, in particular, outperforms a space-based model with a high-level saliency map (S.hl), where object information may be contained in the scene features. The direct comparison between these models demonstrates that the improved performance of object-based models is not solely attributed to the presence of object information. Instead, it is the utilization of objects as perceptual units, enabling attentional mechanisms to act upon them, which leads to more human-like gaze behavior."*

[Cont'd:] *DeepGaze 3 does a pretty good job on static images. To my knowledge, it does not contain a specialised object model, it was just pre-trained on classification and then transfer trained. Wouldn't it work on videos as well? Then, you should obtain clear differences on the dynamic saliency maps. (btw. I'd like to see such data comparing models and humans, not just summary statistics that the model look at some object somewhere.) As this contrast relates to the objectives of the paper, it appears pretty important.*

DeepGaze 3 is an interesting model with an impressive performance at predicting the spatial aspect of scanpaths on static scenes. Making it applicable to dynamic scenes would require its extension towards extracting and processing features from every frame, the capability to perform smooth pursuit, and the prediction of foveation durations. We were already in contact with the authors of DeepGaze 3 prior to the submission of this manuscript, thinking about concrete steps on how to realize it; a comparison, however, is outside the scope of the current manuscript.

If realized, a DeepGaze-like model for scanpath simulation on videos would most likely outperform our models, which have five free parameters only. As comparisons of scanpaths predicted by DeepGaze 3 and the SceneWalk model on static images show, deep learning and mechanistic models can still learn much from each other (Schwetlick, Kümmerer, et al., 2022). In particular, the here investigated distinction between a model using features related to object information in combination with space-based attention (as in *S.hl*) and a model with object-based attentional mechanisms (as in, e.g., *O.ll*) is not possible with machine learning models. In the example of DeepGaze 3, it is not clear to what extent potentially learned object representations are used for target selection.

We elaborate on the concerns about the evaluation methods in response to later comments, where we describe newly introduced evaluation metrics that directly compare human and model behavior.

Schwetlick, L., Kümmerer, M., Engbert, R., & Bethge, M. (2022). DeepGaze vs SceneWalk: what can DNNs and biological scan path models teach each other?. Journal of Vision, 22(14), 3986-3986.

Then there is the inhibition of return (IOR) topic. I'm aware that IOR is a convenient mechanism that saliency map driven models do not always look at the same location. However, the early experimental evidence for IOR found a delay of saccades towards previously fixated locations, not a reduced probability to view previously fixated locations. Here, however, the term is used indiscriminately. Wilming et al., 2013, PLoS CB (sorry for citing my work) gives a reasonable account of this aspect. Given that it is a mechanistic model, here would be the chance for interesting investigations, e.g. does IOR in the correct sense of a delayed of revisits occur? What exactly is the distribution of saccadic vectors in subsequent saccades? How about the timing of fixation durations as a function of saliency of subsequent target? But such questions and timing is not addressed in the study.

By introducing the studies that led to the discovery of IOR in lines 263 ff, we already wanted to clarify that computational models typically do not implement IOR in the historical sense. We make our use of the term now more explicit before describing how we incorporate it in our framework in line 266 ff:

> *"We adopt the common practice in scanpath models to use IOR in terms of an inhibition of previously explored parts of the scene for preventing the continuous return to the same targets with high saliency (Itti and Koch, 2001; Zelinsky, 2008; Bays and Husain, 2012; Schwetlick, Rothkegel, et al., 2020)."*

We agree that investigating IOR in dynamic scenes with a mechanistic modeling approach opens up interesting opportunities. We added a new result section on "*Sequential scanpath evaluation*", where we incorporate the reviewers' suggestions on IOR-related phenomena and timing.

We now analyze the distribution of saccadic vectors in subsequent saccades (new Fig. 8a) starting in line 663 and complement it with a comparison of the prevalence of return saccades starting in line 677.
We investigated foveation times in relation to the relative angle of subsequent saccades to investigate if IOR in the sense of delayed revisits occurs (new Fig. 8b), starting in line 684.
In addition, we also analyzed the frequency and time course of saccades returning to previously explored objects (new Fig. 8c, line 697 ff).
With a total number of only 56 return saccades (Δangle > 178 and Δamplitude < 1.5 dva, as in Wilming et al., 2013) across all videos and observers, the dataset investigated here is unfortunately not suited for a detailed analysis of the timing of fixation durations as a function of saliency in return saccades. We did compare the overall mean saliency (quantified with the low-level saliency map by Molin et al., 2015) of all saccade target positions in the human data (mean 0.123 and standard deviation 0.092) with the target positions of return saccades (0.165 ± 0.102). The different models, most pronounced (as expected) in the *S.ll* model (overall: 0.233 ± 0.104, return: 0.407 ± 0.132), but even the *O.cb* model (overall: 0.104 ± 0.086, return: 0.153 ± 0.111), reproduce this tendency of returning to highly salient locations (*S.hl* with overall: 0.109 ± 0.088, return: 0.149 ± 0.119; *O.ll* with overall: 0.175 ± 0.104, return: 0.246 ± 0.100; *M.ll* with overall: 0.165 ± 0.101, return: 0.209 ± 0.086). Due to the low number of data points, we did not include this analysis in the manuscript. We are, however, planning an in-depth model-based analysis of this and related phenomena (as investigated on static scenes by Wilming et al., 2013) on a large-scale dataset of videos we are currently recording with controlled object and camera movements.

Further, the study investigates eye movements on videos. This is interesting. However, fixations and pursuit movements are lumped together.

We state our reasons for combining fixation and smooth pursuit events now more clearly in line 445 ff:

> *"[...] Across all videos, observers spend 2444 ms on average (median 2200 ms) doing smooth pursuit, compared to 5518 ms (median 5596 ms) of fixation. The classification between smooth pursuit and fixation events is noisy, however, even with state-of-the-art methods (with F1≈ 0.7 for smooth pursuit in Startsev, Agtzidis, and Dorr 2019). Since both keep the current target foveated and are bounded by saccadic decisions, we combined smooth pursuit and fixations to foveation events."*

If the distinction between the two foveation sub-classes were of particular interest, it would be possible to retrospectively separate both event classes in the simulated scanpaths based on the displacement or by setting a velocity threshold.

[Cont'd:] There is no investigation on the influence of motion cues on the triggered eye movements. We do know that motion cues are effective even in static images (Acik et al, 2014, J Vis). How does the present model react to static cues implying motion? How important is the actual motion cues?

As pointed out in the manuscript (line 756 ff) and previously by the reviewer, we did not yet include top-down guidance in our modeling framework. We now added a paragraph explicitly on expectations and semantics in the Discussion, starting in line 762:

> *"Top-down attention in the form of expectations can also play an important role in guiding gaze during free viewing. It has, for example, been shown that already static cues implying motion robustly attract eye movements (Açık, Bartel, and Koenig, 2014).*

We currently have not incorporated any semantic representation of the scene into our framework. Our top-performing model (O.ll) relies solely on low-level features to prioritize between objects and to determine the specific location within that object to direct its gaze. [...]"

Due to the mechanistic design of the models, we would have to explicitly implement a sensitivity to (static) motion cues. The only model component that could potentially pick up on implied motion cues is the TASED-Net high-level saliency map (used in the *S.hl* model) since it is learned from human gaze behavior, but analyzing TASED-Net's behavior in detail is not the focus of this study.

We agree, however, that this is a very interesting question, and we already planned to investigate such expectation/cueing effects. For such a study, however, we must implement an additional "motion expectation" module and must specifically design a dataset to enable targeted hypotheses testing. This is out of the scope of the current manuscript.

[Cont'd:] Or an investigation whether IOR applies to moving objects or locations in allocentric coordinates. ... The study is performed using videos but falls short to deliver clear insights on this aspect.

Whether IOR is object or location-based is an important question, which we now include in the description of IOR in the framework starting from line 291:

"There is, however, also experimental evidence for a reduction of IOR with object movement by Krüger and Hunt (2013), who do not find IOR in object-based coordinates, and other experiments have found IOR in location-based coordinates across object motion before (Müller and Von Mühlenen, 1996; Abrams and Pratt, 2000). To facilitate the comparison between models with a space-based and with an object-based implementation, we define a mixed model M.ll, which is identical to O.ll (cf. Fig. 2) but uses Eq. (1) to calculate the inhibition I instead of Eq. (2)."

We elaborated on the implementation of the *M.ll* model as an example of the flexibility of the *ScanDy* framework in the section on the framework architecture starting in line 421. We fit and evaluate this model using the same procedures as the purely spatial and object-based models. The *M.ll* model allows us to systematically compare the competing assumptions of IOR applying to moving objects or locations in allocentric coordinates. We discuss our findings from the sequential scanpath evaluation in the Discussion, starting in line 880, including:

"[..] We found that an object-based target selection in the decision-making process (module IV) actually has a stronger influence on IOR-related diagnostics than the implementation of the IOR mechanism in module (III). This result again points to the importance of object-based attentional guidance. Reaching a definitive conclusion on whether IOR is bound to objects or locations is not possible due to the limited number of return saccades in the here investigated dataset and the simplicity of our implementation. Evidence from static scenes suggests that instead of merely inhibiting previously fixated locations combination of saccadic momentum and facilitation of return (FOR) best describes human behavior (Wilming et al., 2013). [...]"

The discussion contains a longish paragraph on the insufficiencies of measures comparing trajectories. But this is what the manuscript is about!? Why not use those measures we have? Not offering an alternative is not a solution.

The major analysis rests on the statistics of viewing objects/background, with the variation of detection, inspection, and revisits. Ok, interesting. But it leaves open whether the same objects are detected. The objects are viewed in identical sequence. In how far the results

depend on the level of details of object labelling (e.g. forest or trees). Again, one model variant look at objects for a similar time, does this make it the better model.

We substantially expanded the model evaluation based on the reviewers' suggestions, including a number of sequential metrics which are independent of object segmentations. We expanded the last paragraph (starting in line 839) of the Discussion section on the evaluation of simulated scanpaths accordingly:

> *[...] This approach combines the benefits of string-based methods and dynamic region boundaries while additionally providing information about the extent to which objects are detected, inspected, or returned to. This evaluation is, however, not sensitive to the object identity, meaning a model could detect, inspect and return to objects ignored by human observers and still achieve perfect agreement. We controlled for this by directly comparing the simulated dwell time on each object with the human data. We complemented the functional evaluation, which is based on average viewing metrics, with sequential analyses that explicitly depend on the scanpath dynamics and the previous foveation history. To incorporate the viewing order in our evaluation, we analyzed which objects are first detected when new scenes are explored. We focused this analysis on diagnostics of the IOR effect, including the relative angle of subsequent saccades, the interdependence of foveation duration and this angle, and the frequency and time course of object-based return saccades. This enabled us to investigate the individual contributions of object-based attention on different mechanisms. Additional qualitative evaluations, including the analysis of periods with high inter-observer consistency (Fig. S9), are described in the Supplemental Material.*

Obtaining high-quality segmentations of natural videos on different levels of representation is a very difficult task. State-of-the-art models of video instance segmentation still struggle to produce temporally consistent object IDs, which are crucial for our object-based evaluation. Hence, we did not include a control condition with different levels of detail in the object segmentation, although it is an interesting suggestion. We did, however, add a control condition suggested by Reviewer 3, where we manipulated the scenes such that the scanpaths no longer correlate with the object content (Fig. S8) to the Supplemental Material.

Saccades modelled as instantaneous. Why? On static images I could understand but when you investigate videos it makes a difference whether a saccade last 40ms or 0ms. This simplification appears so unnecessary.

We fully agree with the reviewer that this was an unnecessary omission. We now extended the model to include saccade timings, relying on the main-sequence relationship between saccade amplitude and duration (Eq. 6, line 332), which we now describe together with the implementation of the decision-making module (IV), starting in line 298.

Looking back, there are many interesting bits and pieces but I fail to make up my mind what exactly I've learned. I'd recommend to reconsider what the main story of the study is and then go in depth for that story.

The reviewers' feedback helped us further develop and clearly articulate our main story, that is, the influence of object-based attention on guiding gaze behavior. We hope that the reviewer agrees that – through the additional model comparisons and analyses – our treatment of this story has gained depth and is an interesting contribution to the literature on attentional selection in dynamic scenes.

Reviewer #2: Review for: Objects guide human gaze behavior in dynamic real-world scenes

Roth and colleagues present ScanDy, a novel computational model of human gaze behavior towards dynamic real-world scenes, validated against the VidCom dataset (Li et al., 2011). ScanDy predicts a series of foveation targets (fixations, pursuit) dynamically, i.e. in a time-resolved manner. This is a significant advantage over scanpath models for static scenes or frame-wise predictions of average fixation locations at a group level. It does so based on a modular combination of scene and observer features, allowing for flexible hypothesis testing.

The authors combine modules capturing salience, foveation and inhibition of return with a drift diffusion model of a decision process, leading to five free parameters. These are fitted to a human distribution of foveation durations and saccade amplitudes and then evaluated against human hold-out data (gaze towards a separate set of videos). Both, human behavior and model predictions are stable with respect to these basic parameters.

Importantly, model predictions are further compared to human behavior concerning the predicted proportion of background foveations versus three types of object foveations: Detection foveations (landing on a given object for the first time), Inspection foveations (saccades within a given object), and Returns (returns to a previously foveated object). This is done for four models: Two that base their predictions on locations (i.e. pixel-based foveation, IOR and decisions) and two that consider objects for foveation, IOR and the decision process. A model combining pixel-based low-level salience with object based foveation, IOR and decisions clearly outperforms the rest in recapitulating the proportions of Background / D/I/R foveations of human observers. The authors conclude that human gaze behavior towards dynamic scenes is (at least to a significant degree) guided by objects.

Strengths:

This paper is excellent. It is exceptionally well written, with a comprehensive literature review and thoughtful discussion. ScanDy is a substantial advance over previous models in the right direction (capturing natural gaze behavior) and promises to be a valuable tool for the community. Its modular architecture provides an avenue for testing numerous model features and hypotheses beyond the ones presented here. A particularly appealing feature is the accessibility of the code, including a Colab instantiation of an interactive notebook. Clearly, much effort and care went into this, and the result is impressive! The model also provides convincing evidence for the tested hypothesis that objects guide human free viewing to dynamic scenes beyond frame-wise observer averages.

Suggestions:

1) The authors evaluate model performance against the overall proportions of background / D/I/R foveations across all hold-out videos. The main message from the model comparisons seems to be that models lacking object-based information predict too few object foveations, providing convincing evidence that objects matter for foveations in dynamic scenes.

Nevertheless, it would be interesting to further challenge the model(s) by addressing the question of _which_ objects are being detected, inspected and returned to. The current evaluation scheme is agnostic towards this, so a model could (theoretically) achieve perfect agreement, even if there was zero overlap between the objects foveated by humans and the model. One way of going beyond this in the DIR framework would be to quantify DIR ratios (or absolute dwell times) separately for each object and average absolute deviations between predictions and observations across objects. This metric would complement the authors'

approach and provide an indication of the degree to which a model predicts _which_ objects attract D/I/R foveations.

We thank the reviewer for the great summary and kind comments. We extended the functional scanpath evaluation based on the DIR framework by quantifying the total dwell times for individual objects, starting in line 622. Instead of averaging the absolute deviations, we hope to have found a more intuitive measure to compare model data with humans by plotting the average dwell time of simulated model scanpaths as a function of the total average dwell time across human observers for each object (Fig. 7). We fit a regression line for each model and compare the explained variance, slope, and intercept. While this could be done for each DIR category individually, we found that this would not substantially add to the combined information of Figs. 5, 6, and 7 of the revised manuscript.

2) The modular model architecture encourages readers to build upon it and explore their own ideas for extending or changing the model (some of which the authors foreshadow, such as perisaccadic shifts of attention). This is a fantastic feature and important to bear in mind regarding all questions about model specifications.
While hoping not to fall into the 'why don't you do it my way' trap, I would like to point out that I find the choice of salience maps for the object and location-based models difficult to understand. Doesn't the high-level salience map introduce object-based information (or a good proxy for that) to the location-based model? And why is the center-bias approach limited to the object-based models? Both these choices seem counterintuitive to me. If there are good reasons for them, it would be helpful to receive (further) explanation (apologies if I missed something obvious). If not, it may be more intuitive to leave out object-based information at this stage of the model altogether and keep the salience maps identical between the location- and object-based models (i.e. low-level and center bias maps for both, the location and object based models).

We substantially revised parts of the introduction (starting in line 148) to motivate more clearly why we chose these models to investigate the role of object-based attention on guiding gaze behavior:

> *"[...] For this, we analyzed different model implementations within the ScanDy framework: The first model is purely space-based and uses a biologically plausible low-level saliency map (S.ll model). We compared this with an object-based model which uses the same low-level saliency map but where attentional mechanisms, like the computation of visual sensitivity, inhibition of return, and the saccade target selection (cf. Sec. ScanDy in Methods), are based on objects (O.ll model). Comparing only these two models may, however, be unfair due to the additional scene information (in the form of object segmentation masks) available to the object-based model. Therefore, we introduced a space-based model with a high-level saliency map (S.hl model) as an additional comparison, where object information may be contained in the scene features, but the attentional mechanisms acting on them are purely spatial. We also investigated an object-based model which does not use any saliency information but only the center bias (O.cb model) to better understand the interaction of object-based attention and saliency. Lastly, we explored the hypothesis of a spatial inhibition of return mechanism applying to locations in allocentric coordinates (Krüger and Hunt, 2013) together with an object-based visual sensitivity and target selection in a "mixed" model that uses the low-level saliency map (M.ll model)."*

Hence, instead of reducing the available features for the *S.ll* model (a space/location-based model with center bias only would have no information about the scene at all), we wanted to make the comparison more challenging for the *O.ll* model. As described in detail in response to Reviewer 1 and stated more clearly throughout the revised version of the manuscript, by comparing *S.hl* and *O.ll* directly, we demonstrate the effect of object-based attentional mechanisms in contrast to object-related feature information and space-based attention. We make this now explicit in the Discussion, starting in line 875:

> *"[...] The O.ll model, in particular, outperforms a space-based model with a high-level saliency map (S.hl), where object information may be contained in the scene features. The direct comparison between these models demonstrates that the improved performance of object-based models is not solely attributed to the presence of object information. Instead, it is the utilization of objects as perceptual units, enabling attentional mechanisms to act upon them, which leads to more human-like gaze behavior."*

Minor:
1) The zero-lag of transitions between foveations causes excess total foveation times in the models. Is there a reason for not including a simple lag, scaling with amplitude to avoid this problem? The authors suggest more sophisticated extensions in this direction and I see how this would be more of a follow-up (see above). Still, I wonder whether a simpler solution wouldn't go a long way to alleviate this problem and straightforward to implement in the existing model? Either way, this is just a suggestion and I would like to leave it to the authors whether to incorporate this or not.

We incorporated this valuable recommendation in the modeling framework and describe the implementation as part of the decision-making module (IV), starting in line 298 (see Eq. 6, in line 332), and updated the discussion of a more sophisticated extension (line 745 f) accordingly to:

> *"In this work, we assume a simple linear relationship between the amplitude and duration of saccades (Collewijn, Erkelens, and Steinman, 1988) and that saccades always precisely reach their target. [...]"*

2) The authors mention that objects in the context of their model are restricted to humans, animals and vehicles. It may make sense to highlight this more prominently and discuss the fact that all of these typically are moving objects. Cursory readers should be aware of the fact that 'background' in this context includes all sorts of inanimate objects.

We now emphasize our definition of objects and background again in the last paragraph of the Discussion, starting in line 901.

> *"We define humans, animals, and vehicles as objects, which are most relevant to the content in the here investigated scenes and can potentially show object motion. [...]"*

3) I'm wondering: would it make any difference to drop the modelling of foveational eye movements?

This is an interesting point. As now briefly stated in the description of the Gaze update (V) in line 350 ff:

This Brownian motion, in addition to making the generated data appear visually more like human eye tracking data, also contributes to the variability in the simulated scanpaths by shifting the center of the gaze-dependent visual sensitivity (module II).

The influence is, however, not systematic and does not significantly affect our evaluation metrics.

4) Figure S7 (which I found informative) could be added as additional panels to Figure 5.

We agree that the time courses of the functional decomposition are interesting and included them in Fig. 5. We especially found the initial peak of detection events informative for the model comparison, which we now describe in detail, starting in line 556.

5) I'm not sure, I understand Figures S3-6. Does the diagonal show inter-individual parameter consistency? If so, could the lacking convergence for IOR decay point to genuine inter-observer variance?

We apologize for the confusing usage of the term *individual* in the caption of Figures S3-6. In this context, it did not refer to individual human observers but to the "individuals" (i.e., model parameter configurations) in the population of the last generation in the evolutionary optimization. We fit the parameters on the combined data of all scanpaths of videos in the test set. Therefore, the lacking convergence here does not reflect inter-observer variance but shows that the fitness function does not fully constrain the parameter for the IOR decay, such that models can have different parameter values for the IOR decay but still perform equally well. The revised caption of Fig. S3 now reads:

> ***Figure S3.*** *Visualization of the parameter space of the space-based model with low-level features (S.ll). The diagonal shows for each parameter the distribution across the last generation of the evolutionary optimization process. The last generation contains the 32 model parameter configurations with the highest fitness (as defined in Eq. (8) in the main paper). Other panels show how pairs of parameters relate to each other.*

6) I'm glad the authors found the D/I/R classification helpful. We have just published a preprint that introduces the approach and gives more detail than the cited abstract: https://psyarxiv.com/bqfdy/ Please note we now refer to 'Returns' instead of 'Revisits' to stay more consistent with the wider literature.

We changed the citation from the VSS abstract to the preprint and updated all mentions of "Revisits" to "Returns" in the manuscript.

Reviewer #3:
The question of eye guidance has been receiving a lot of attention in the modelling literature, probably starting from applying Koch & Ullman's salience map to natural scenes in the late 1990s and not ending with current DNN-based models. Somewhat surprisingly, there have been largely distinct strands of modelling, such that it is a worthwhile endeavor to combine these into a single framework. Consequently, while none of the components of ScanDy may be groundbreaking in and by itself, the integration of many components certainly has a value for the field. This integration is performed by authors with the required in-depth knowledge of the field (and the various modelling strands). Hence, the paper in addition to an interesting model also provides a good overview on the state of the field, such that I would consider the manuscript potentially of interest also for the broad readership and therefore potentially suitable for PLoS CB.
I have one major concern, however:
(1) From the abstract I would have expected a direct comparison of scanpaths to human data (when reading it again, I understand that "including the proportion…" is meant as an exhaustive list). However, this happens only on the basis of summary statistics (those mentioned after "including…"). The authors put a lot of effort in the Discussion why they refrain from direct comparisons, but I don't think these are fully convincing:

We have substantially extended on the scanpath evaluations with metrics that complement the previously reported summary statistics (see also our response to Reviewer 1 and Reviewer 2). In the functional evaluation, we now directly compare the total object dwell time across human observers with the model predictions (Fig. 7, starting in line 627). In a new section on the sequential scanpath evaluation, we go beyond summary statistics and explicitly investigate which objects are detected first and explore the effect of the fixation history on upcoming saccades. We thank the reviewer for the four additional suggestions, out of which we implemented (a) and (c). We briefly discuss why we prefer these over the other two ideas in the following.

a. The authors could focus on periods/epochs of high inter-observer consistency with respect to the scanpath (e.g., nearly all observers looking at the same region at some point and then see how model and observers evolve from there).

In the here investigated VidCom dataset, all trials start with the observers fixating on the center of the screen. Following the reviewers' suggestion, we analyzed which objects are detected first by humans (starting in line 649) as part of the sequential scanpath evaluation.
In addition, we identified prominent peaks in coherence between observers (measured by the Normalized Scanpath Saliency (NSS) score as in Dorr et al., 2010) and qualitatively compared representative examples in Fig. S9 in the Supplemental Material, which we think provides a good intuition about the differences and shortcomings of the different models. Further quantifying these periods is, however, challenging. Potential difficulties include a principled selection of peaks in the videos (highest peak in each video [except the starting position] vs. absolute threshold) and the influence of previous scanpath history.

b. They could apply their model to static scenes and compare the prediction to models tailored for this purpose with the metrics they discuss.

It is possible to apply the here presented framework to static images in a straightforward way by treating images as static videos, i.e., feeding the same image as the frame into the model at each time step. Yet, motion is an important cue, and it is possible that models for ecologically more relevant scenes should be designed differently from those for static scenes. Of course, a perfect model of human gaze behavior should generalize to all scenes, but such a model would most likely be more complicated than the models proposed here, which include five free parameters only. Hence, we would prefer to concentrate our efforts on evaluating the models on dynamic scenes.

c. They could construct a control condition, where the distributions of figure 4 are still reproduced, but the scanpath statistics would differ (e.g., reversing or shuffling the video for computing the scanpath evaluation metrics)

We agree that this is a useful control and now introduce it in the functional analysis, starting in line 567:

> *We introduced a control condition where we manipulated the scenes such that the scanpaths no longer correlate with the object content. For this, we reversed the video frames in time and mirrored them on the x-axis and on the y-axis while keeping the scanpaths of the models and human observers unchanged. As expected, this manipulation leads to both the human and model scanpaths predominantly exploring the background and causes the previously described effects to disappear (we show the equivalent of Fig. 5 for this control condition in Fig. S8 in the Supplemental Material).*

d. They could include comparisons to static scanpath models (e.g., show the static model a frame from the video and apply the static prediction to the next 1s of the video).

This is an interesting idea that is similar to the approach by Tangemann et al. (2020), who compare their predicted fixation probability density for static images on dynamic scenes with that of video saliency models by applying it to each frame independently. While this works surprisingly well for saliency models, scanpath models like ours depend much more on the previous history. Therefore, it would not be fruitful to predict a scanpath on the 2nd second of a stop-motion video independent of the scanpath from the 1st. We feel that adopting the static models accordingly would be outside the scope of this work.

Tangemann, M., Kümmerer, M., Wallis, T. S., & Bethge, M. (2020). Measuring the Importance of Temporal Features in Video Saliency. In European Conference on Computer Vision (pp. 667-684).

There are probably more options, but prediction beyond approximately reproducing four coarse categories would strengthen the paper.

Based on the suggestions of all reviewers, we updated our approach to the evaluation of scanpaths in the last paragraph of the Discussion, starting in line 849, to:

> *"[...] We complemented the functional evaluation, which is based on average viewing metrics, with sequential analyses that explicitly depend on the scanpath dynamics and previous foveation history. To incorporate the viewing order in our evaluation, we analyzed which objects are first detected when new scenes are explored.*
> *We focused this analysis on diagnostics of the IOR effect, including the relative angle of subsequent saccades, the interdependence of foveation duration and this angle,*

and the frequency and time course of object-based return saccades. This enabled us to investigate the individual contributions of object-based attention on different mechanisms. Additional qualitative evaluations, including the analysis of periods with high inter-observer consistency (Fig. S9), are described in the Supplemental Material."

(2) One additional reason why I am worried about the scanpath evaluation is that to my understanding the assignment of Background, Detection, Inspection and Revisit heavily relies on the definition of an object. Could this bias in favor of object-based models.

The reviewer raises an important point. As described in the Discussion section on the *Evaluation of simulated scanpaths* (now line 843 ff):

> *"Importantly, we extended the three originally proposed categories (Detection, Inspection, and Return) by a fourth Background category. This addition effectively avoids a biased evaluation that would only consider foveation events on objects."*

While it is true that it would be easier to fit the object-based models to these object-based metrics, since the parameters map naturally, it is critical that we only optimize the models to reproduce the basic scanpath statistics. We emphasize this now once more in the Discussion, starting in line 864:

> *"[...] The object-based model's ability to replicate a wide range of functional and sequential aspects of human scanpaths is especially surprising, considering that its parameters were fitted solely to match the general distributions of foveation durations and saccade amplitudes."*

We do, however, fully agree that it is good to have additional evaluation metrics that are independent of the object definitions. The newly-added analyses—including the sequential scanpath evaluation (starting in line 644), the qualitative scanpath analyses, and the introduced control condition using misplaced object masks—serve this purpose. We hope that, in sum, the now-presented evidence in favor of the *O.II* model is convincing.

Other comments (without particular order)

(3) In the context of extracting scene layout faster than the objects, the early work by Schyns and Oliva [e.g., 1] on hybrid scenes might be worth mentioning.

We added the reference in line 201 as:

> *"Early work by Schyns and Oliva (1994) shows that humans are able to extract the layout of a novel scene and understand its meaning within less than 300 ms of a single fixation. [...]"*

(4) Given that the authors find their O.II model to be the best performing model and this is a central point of the present work, I would recommend to discuss the relation to the work by Stoll and colleagues [2]. These authors showed experimentally that a combination of hand-crafted object outlines and a central-bias like mechanism per object [3] outperforms other models, and – probably more relevant for the present discussion – also demonstrated that the selection among objects is then based on salience. While the approaches are very different to the present work, it seems that the conclusions regarding fixation probability in this work are similar to the conclusions on scan paths in the present work, such that these results nicely complement each other.

We thank the reviewer for this suggestion and agree that the work by Stoll et al. (2015) is relevant. We added the following paragraph in line 867 of the Discussion section:

“Specifically, we find that the O.ll model, which prioritizes objects based on low-level saliency information, performs best across metrics. This is consistent with results from scene viewing in static images, where Stoll et al. (2015) show that, when taking the preferred viewing location close to the center of the object into account, object outlines outperform models of early salience. Stoll et al. (2015) additionally use statistical models to estimate the impact of object properties on the fixation probability, finding that attention is primarily guided to objects and objects with higher low-level saliency are preferentially selected. Together with related work by Nuthmann, Schütz, and Einhäuser (2020), which also shows salience-based object prioritization, this emphasizes that previous work – while using complementary approaches – has come to the same conclusions as presented here.”

(5) Figure 4b: Even considering the “artifact” (l. 431), is it obvious that the reduced number of small saccades is all due to technical issues? Or might it be more appropriate to fit a Gamma-distribution, which for large amplitudes will approach the exponential?

It is correct that a Gamma-distribution fits the histogram of saccade amplitudes well. We replaced the exponential fit in Fig. 4b with a Gamma-distribution and adapted the description in the manuscript accordingly.

(6) Given the relevance of smooth pursuit mentioned at several points: how abundant are smooth pursuit epochs in the dataset? Could the authors give an approximate fraction , like in XXX of the videos we observe smooth pursuit longer than – say – 300ms?

We added the following information to the Methods section *Dataset*, starting in line 443:

When taking the average smooth pursuit duration (as classified using the algorithm by Startsev, Agtzidis & Dorr, 2019) across observers for each video (all 10000 ms long), the smallest fraction of smooth pursuit we observe in a video is 543 ms (scene: park01). Across all videos, observers spend 2444 ms on average (median 2200 ms) doing smooth pursuit, compared to 5518 ms (median 5596 ms) of fixation.
